# Supplementary material for: Identification of Freezing-Responsive microRNAs and Their Targets in Chinese Jujube by Small RNA and Degradome Sequencing
Source: Int J Mol Sci. 2026 Sep 4;27(17):7912. doi: 10.3390/ijms27177912 (PMC13566890; doi:10.3390/ijms27177912)
Supplement: Supplementary file 1 [file ijms-27-07912-s001.zip › Supplementary Material S2/novel_miR_181.pdf]

Provisional ID	: novel_miR_181
Score total	: 1
Score for star read(s)	: -1.3
Score for read counts	: 0
Score for mfe	: 0.7
Score for randfold	: 1.6
Score for cons. seed	:
Total read count	: 1039
Mature read count	: 887
Loop read count	: 0
Star read count	: 152

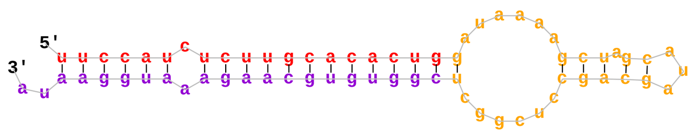

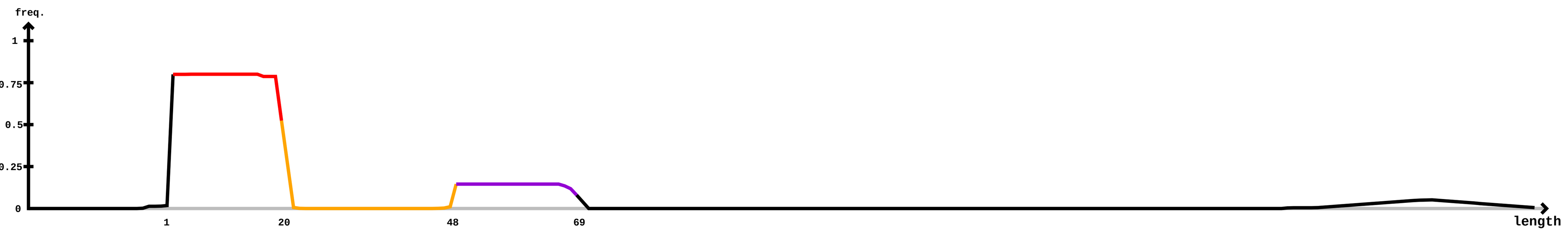

Mature Star

```
5'- gcucucaugguuuuaugguauauuccaucucuugcacacuggauaaaagcuagcauagcagccucggcucggugugcaagaaauggaauauauuacugaaucaugcuuuggagcaagaauauucggauacaguguaucuacuuucuuccuuaaucacugauucaagguaaaagagauuucuccaucuuccuucuuugcucccucaaaugaagcaaaaaaaaaaaaaaaaaaaaaaaaaaaaaaaaaaaaaaa -3' obs
    gcucucaugguuuuaugguauauuccaucucuugcacacuggauaaaagcuagcauagcagccucggcucggugugcaagaaauggaauauauuacugaaucaugcuuuggagcaagaauauucggauacaguguaucuacuuucuuccuuaaucacugauucaagguaaaagagauuucuccaucuuccuucuuugcucccucaaaugaagcaaaaaaaaaaaaaaaaaaaaaaaaaaaaaaaaaaaaaaa exp
    (((..((((((((..((((((((((((.((((((((((((......(((.((...)))))......))))))))))))).)))))))))))))..))))))))....(((((((((.....((((..((...((((.((((...((((((((...)))).)))))..)))))))))..))..))))...))).)))))))).........)))......................................
```

alignment	reads	mm	sample
................guauauuccaucucuugcac	1	0	LT-1
................guauauuccaucucuugcac	1	0	LT-3
.................uauauuccaucucuugcac	1	0	JCK-1
.................uauauuccaucucuugcac	8	0	LCK-3
.................uauauuccaucucuugcac	2	0	LT-3
.................uauauuccaucucuugcac	2	0	JCK-2
...................uauuccaucucuugcacacug	1	0	LT-3
....................auuccaucucuugcacacugg	1	0	JCK-1
....................auuccaucucuugcacacugg	1	0	JCK-2
....................auuccaucucuugcacacugg	2	0	JCK-3
.....................uuccaucucuugcacacu	7	0	JCK-1
.....................uuccaucucuugcacacu	9	0	JCK-2
.....................uuccaucucuugcacacG	1	1	LT-1
.....................uuccaucucuugcacacu	12	0	JCK-3
.....................uuccaucucuugcacacu	19	0	LT-1
.....................uuccaucucuugcacacu	75	0	LCK-3
.....................uuccaucucuugcacacu	32	0	LCK-2
.....................uuccaucucuugcacacu	51	0	LT-2
.....................uuccaucucuugcacacu	41	0	LT-3
.....................uuccaucucuugcacacu	3	0	JT-3
.....................uuccaucucuugcacacu	39	0	LCK-1
.....................uuccaucucuugcacacu	6	0	JT-2
.....................uuccCucucuugcacacu	1	1	LT-2
.....................uuccaucucuugcCcacu	1	1	LT-1
.....................uuccaCcucuugcacacug	1	1	LCK-3
.....................uuccaucucuugcacacug	10	0	LT-1
.....................uuccaucucuugcacacug	41	0	JT-1
.....................uuccaucucuugcacacug	49	0	LT-2
.....................uuccCucucuugcacacug	1	1	JCK-3
.....................uuccaucucuugcacacug	5	0	JCK-1
.....................uuccCucucuugcacacug	1	1	LT-3
.....................uuccaucucuugcacacug	5	0	JT-2
.....................uuccaucucuugcacacug	23	0	JCK-2

```
                      Mature                                               Star
gcucucaugguuuuaugguauauuccaucucuugcacacuggauaaaagcuagcauagcagccucggcucggugugcaagaaauggaauauauuacugaaucaugcuuuggagcaagaauauucggauacaguguaucuacuuucuuccuuaaucacugauucaagguaaaagagauuucuccaucuuccuucuuugcucccucaaaugaagcaaaaaaaaaaaaaaaaaaaaaaaaaaaaaaaaaaaaa
......................uuccaucucuugcacacug.  29  0  LT-3
......................uuccaucucuCgcacacug.  1  1  JCK-3
......................uuccaucucuugcCcacug.  1  1  LT-2
......................uuccaucucuugcacacug.  10  0  JT-3
......................uuccaucucuugcacacug.  93  0  LCK-3
......................uuccaucucuugcacacug.  8  0  JCK-3
......................uuccaucucuugcacacugU.  1  1  JCK-1
......................uuccaucucuugcacacugg.  20  0  JCK-3
......................uuccaucucuugcacacugg.  14  0  JT-1
......................uuccaucucuugcacacugg.  17  0  JCK-2
......................uucNaucucuugcacacugg.  1  1  JCK-1
......................uuccaucucuugcacacugg.  34  0  LCK-1
......................uuccaucucuugcacacugg.  5  0  JT-3
......................uuccaucucuugcacacugg.  19  0  LCK-2
......................uuccaucucuugcacacugg.  62  0  LCK-3
......................uuccaucucuugcacacugg.  13  0  JCK-1
......................uuccaucucuugcacacugg.  50  0  LT-3
......................uuccaucucuugcacacugg.  5  0  JT-2
......................uuccaucucuugcacacugg.  32  0  LT-1
......................uuccaucucuugcacacugg.  27  0  LT-2
......................uuccaucucuugcacacuggC.  1  1  LT-3
......................uuccaucucuugcacacugga.  2  0  JCK-2
......................uuccaucucuugcacacugga.  1  0  JCK-3
......................uuccaucucuugcacacugga.  1  0  JCK-1
......................uuccaucucuugcacacuggau.  1  0  LT-3
.........................caucucuugcacacugga.  1  0  LT-2
..................................................................gcucggugugcaagaaauggaaua.  1  0  LT-3
...................................................................cucggugugcaagaaauggaau.  2  0  JCK-2
....................................................................ucggugugcaagaaauggaa.  4  0  LCK-3
....................................................................ucggugugcaagaaauggaau.  3  0  LT-3
....................................................................ucggugugcaagaaauggaau.  2  0  LT-2
.....................................................................cggugugcaagaaaugga.  1  0  JCK-2
.....................................................................cggugugcaagaaaugga.  1  0  LT-1
.....................................................................cggugugcaagaaaugga.  6  0  LT-3
.....................................................................cggugugcaagaaaugga.  1  0  JCK-1
.....................................................................cggugugcaagaaaugga.  3  0  LT-2
.....................................................................cggugugcaagaaauggaa.  1  0  LT-3
.....................................................................cggugugcaagaaauggaa.  1  0  LT-2
.....................................................................cggugugcaagaaauggaa.  6  0  JT-3
.....................................................................cggugugcaagaaauggaa.  5  0  JT-2
.....................................................................cggugugcaagaaauggaU.  2  1  LCK-3
.....................................................................cggugugcaagaaauggaau.  20  0  LCK-3
.....................................................................cggugugcaagaaauggaau.  3  0  JCK-3
.....................................................................cggugugcaagaaauggaau.  5  0  LT-3
.....................................................................cggugugcaagaaauggaau.  3  0  LT-1
.....................................................................cggugugcaagaaauggaau.  4  0  LT-2
.....................................................................cggugugcaagaaauggaau.  1  0  JCK-2
.....................................................................cggugugcaagaaauggaauU.  3  1  LT-3
.....................................................................cggugugcaagaaauggaaua.  8  0  JCK-3
.....................................................................cggugugcaagaaauggaaua.  5  0  JCK-1
.....................................................................cggugugcaagaaauggaaua.  5  0  LT-2
.....................................................................cggugugcaagaaauggaaua.  5  0  JCK-2
.....................................................................cggugugcaagaaauggaaua.  5  0  LT-1
.....................................................................cggugugcaagaaauggaaua.  5  0  LT-3
.....................................................................cggugugcaagaaauggaaua.  46  0  LCK-3
.....................................................................cggugugcaagaaauggaauau.  7  0  LCK-3
...............................................................................................................................................................................................................ugGagcaaaaaaaaaaaaaaaa.  1  1  JCK-3
...............................................................................................................................................................................................................ugGagcaaaaaaaaaaaaaaaaa.  1  1  JCK-2
...............................................................................................................................................................................................................ugaaUcaaaaaaaaaaaaaaaaaa.  1  1  JCK-2
...............................................................................................................................................................................................................ugGagcaaaaaaaaaaaaaaaaaaaa.  1  1  JCK-2
................................................................................................................................................................................................................gaGgcaaaaaaaaaaaaaaaaaaa.  1  1  LT-1
....................................................................................................................................................................................................................caaaaaaaaaaaaaaaaaaaaaaaUaa.  1  1  JT-1
.....................................................................................................................................................................................................................aNaaaaaaaaaaaaaaaaa.  19  1  JCK-3
.....................................................................................................................................................................................................................aaaaaaaaaaaaaaNaaaa.  19  1  LCK-1
.....................................................................................................................................................................................................................aaaaaaaaaaaaaNaaaaaaa.  17  1  LCK-1
......................................................................................................................................................................................................................aNaaaaaaaaaaaaaaaaa.  19  1  JCK-3
......................................................................................................................................................................................................................aaaaaaaaaaaaaaNaaaa.  19  1  LCK-1
......................................................................................................................................................................................................................aaaaaaaaaaaaaNaaaaaaa.  17  1  LCK-1
.......................................................................................................................................................................................................................aNaaaaaaaaaaaaaaaaa.  19  1  JCK-3
.......................................................................................................................................................................................................................aaaaaaaaaaaaaaNaaaa.  19  1  LCK-1
```

```
                            Mature                                                          Star
gcucucaugguuuuaugguauauuccaucucuugcacacuggauaaaagcuagcauagcagccucggcucggugugcaagaaauggaauauauuacugaaucaugcuuuggagcaagaauauucggauacaguguaucuacuuucuuccuuaaucacugauucaagguaaaagagauuucuccaucuuccuucuuugcucccucaaaugaagcaaaaaaaaaaaaaaaaaaaaaaaaaaaaaaaaaaaaa
.......................................................................................................................................................................................................................aaaaaaaaaaaaaNaaaaaaa..............   17   1   LCK-1
........................................................................................................................................................................................................................aaaaaaaaaaaaaaNaaaa...............   19   1   LCK-1
........................................................................................................................................................................................................................aNaaaaaaaaaaaaaaaaa...............   19   1   JCK-3
........................................................................................................................................................................................................................aaaaaaaaaaaaaNaaaaaaa.............   17   1   LCK-1
.........................................................................................................................................................................................................................aNaaaaaaaaaaaaaaaaa..............   19   1   JCK-3
.........................................................................................................................................................................................................................aaaaaaaaaaaaaaNaaaa..............   19   1   LCK-1
.........................................................................................................................................................................................................................aaaaaaaaaaaaaNaaaaaaa............   17   1   LCK-1
..........................................................................................................................................................................................................................aaaaaaaaaaaaaaNaaaa.............   19   1   LCK-1
..........................................................................................................................................................................................................................aNaaaaaaaaaaaaaaaaa.............   19   1   JCK-3
..........................................................................................................................................................................................................................aaaaaaaaaaaaaNaaaaaaa...........   17   1   LCK-1
...........................................................................................................................................................................................................................aNaaaaaaaaaaaaaaaaa............   19   1   JCK-3
...........................................................................................................................................................................................................................aaaaaaaaaaaaaaNaaaa............   19   1   LCK-1
...........................................................................................................................................................................................................................aaaaaaaaaaaaaNaaaaaaa..........   17   1   LCK-1
............................................................................................................................................................................................................................aaaaaaaaaaaaaaNaaaa...........   19   1   LCK-1
............................................................................................................................................................................................................................aNaaaaaaaaaaaaaaaaa...........   19   1   JCK-3
............................................................................................................................................................................................................................aaaaaaaaaaaaaNaaaaaaa.........   17   1   LCK-1
.............................................................................................................................................................................................................................aNaaaaaaaaaaaaaaaaa..........   19   1   JCK-3
.............................................................................................................................................................................................................................aaaaaaaaaaaaaaNaaaa..........   19   1   LCK-1
.............................................................................................................................................................................................................................aaaaaaaaaaaaaNaaaaaaa........   17   1   LCK-1
..............................................................................................................................................................................................................................aaaaaaaaaaaaaaNaaaa.........   19   1   LCK-1
..............................................................................................................................................................................................................................aNaaaaaaaaaaaaaaaaa.........   19   1   JCK-3
..............................................................................................................................................................................................................................aaaaaaaaaaaaaNaaaaaaa.......   17   1   LCK-1
...............................................................................................................................................................................................................................aaaaaaaaaaaaaaNaaaa........   19   1   LCK-1
...............................................................................................................................................................................................................................aNaaaaaaaaaaaaaaaaa........   19   1   JCK-3
...............................................................................................................................................................................................................................aaaaaaaaaaaaaNaaaaaaa......   17   1   LCK-1
................................................................................................................................................................................................................................aaaaaaaaaaaaaaNaaaa.......   19   1   LCK-1
................................................................................................................................................................................................................................aNaaaaaaaaaaaaaaaaa.......   19   1   JCK-3
................................................................................................................................................................................................................................aaaaaaaaaaaaaNaaaaaaa.....   17   1   LCK-1
.................................................................................................................................................................................................................................aNaaaaaaaaaaaaaaaaa......   19   1   JCK-3
.................................................................................................................................................................................................................................aaaaaaaaaaaaaaNaaaa......   19   1   LCK-1
.................................................................................................................................................................................................................................aaaaaaaaaaaaaNaaaaaaa....   17   1   LCK-1
..................................................................................................................................................................................................................................aaaaaaaaaaaaaaNaaaa.....   19   1   LCK-1
..................................................................................................................................................................................................................................aNaaaaaaaaaaaaaaaaa.....   19   1   JCK-3
..................................................................................................................................................................................................................................aaaaaaaaaaaaaNaaaaaaa...   17   1   LCK-1
...................................................................................................................................................................................................................................aaaaaaaaaaaaaaNaaaa....   19   1   LCK-1
...................................................................................................................................................................................................................................aNaaaaaaaaaaaaaaaaa....   19   1   JCK-3
...................................................................................................................................................................................................................................aaaaaaaaaaaaaNaaaaaaa..   17   1   LCK-1
....................................................................................................................................................................................................................................aNaaaaaaaaaaaaaaaaa...   19   1   JCK-3
....................................................................................................................................................................................................................................aaaaaaaaaaaaaaNaaaa...   19   1   LCK-1
....................................................................................................................................................................................................................................aaaaaaaaaaaaaNaaaaaaa.   17   1   LCK-1
.....................................................................................................................................................................................................................................aNaaaaaaaaaaaaaaaaa..   19   1   JCK-3
.....................................................................................................................................................................................................................................aaaaaaaaaaaaaaNaaaa..   19   1   LCK-1
.....................................................................................................................................................................................................................................aaaaaaaaaaaaaNaaaaaaa   17   1   LCK-1
......................................................................................................................................................................................................................................aaaaaaaaaaaaaaNaaaa.   19   1   LCK-1
......................................................................................................................................................................................................................................aNaaaaaaaaaaaaaaaaa.   19   1   JCK-3
.......................................................................................................................................................................................................................................aaaaaaaaaaaaaaNaaaa   19   1   LCK-1
.......................................................................................................................................................................................................................................aNaaaaaaaaaaaaaaaaa   19   1   JCK-3
```
